# Phylogeographic Analysis of Clade 2.3.4.4b H5N1 in Serbia Reveals Repeated Introductions and Spread Across the Balkans

**DOI:** 10.3390/pathogens14070636

**Published:** 2025-06-25

**Authors:** Sofija Šolaja, Dimitrije Glišić, Ljubiša Veljović, Ivan Milošević, Emilija Nićković, Jakov Nišavić, Vesna Milićević

**Affiliations:** 1 Department of Virology, Institute of Veterinary Medicine of Serbia, Janisa Janulisa 14, 11000 Belgrade, Serbia; dimitrije.glisic@nivs.rs (D.G.); ljubisa.veljovic@nivs.rs (L.V.); vesna.milicevic@nivs.rs (V.M.); 2 Department of Histology and Embryology, Faculty of Veterinary Medicine, University of Belgrade, Bulevar Oslobođenja 18, 11000 Belgrade, Serbia; ikavet@vet.bg.ac.rs (I.M.); emilijanickovic@vet.bg.ac.rs (E.N.); 3 Department of Microbiology, Faculty of Veterinary Medicine, University of Belgrade, Bulevar Oslobođenja 18, 11000 Belgrade, Serbia; jakovmoni@vet.bg.ac.rs

**Keywords:** HPAI, influenza A virus, BEAST X, phylogeography, domestic poultry, Serbia

## Abstract

Highly pathogenic avian influenza (HPAI) H5N1 continues to pose a major threat to animal and public health. Since its emergence, clade 2.3.4.4b has become the dominant global lineage, associated with widespread outbreaks in Europe. This study aimed to characterise the H5N1 strains detected in backyard chickens in Serbia and investigate their phylogenetic and phylogeographic relationships with historical and currently circulating strains in Serbia and the broader region. Samples collected in 2024 were tested by qRT-PCR, and positive samples were propagated in MDCK cells. Whole genome sequencing of isolated virus was performed using the MinION Mk1C platform (ONT, Oxford, UK). Bayesian phylogenetic and phylogeographic analyses were conducted using BEAST X and SPREAD3 v.9.6. The results revealed three independent introductions of H5N1 into Serbia between 2020 and 2024. The spatiotemporal diffusion patterns confirmed both north–south and west–east viral spread from Northern and Central Europe into the Balkans. Additionally, Serbia was identified as a critical transit and divergence point in the regional transmission network, highlighting its role in the spread of H5N1 between Western and Eastern Europe. These findings underscore the importance of continued genomic surveillance in both domestic and wild bird populations to better understand and reduce HPAI spread.

## 1. Introduction

Highly pathogenic avian influenza (HPAI) virus remains one of the most significant threats to both public and animal health [1]. Influenza viruses possess a segmented RNA genome comprising eight segments that encode ten proteins: haemagglutinin (HA), neuraminidase (NA), matrix proteins (M1 and M2), non-structural proteins (NS1 and NS2), nucleoprotein (NP), and three polymerase proteins—polymerase basic 1 (PB1), polymerase basic 2 (PB2), and polymerase acidic (PA). As of 2024, the virus is classified within the family *Orthomyxoviridae*, genus *Alphainfluenzavirus*, and species *Alphainfluenzavirus influenzae* [2,3]. The H5Nx subtype of influenza A, one of the most significant strains today, first emerged in Asia approximately two decades ago. Since then, it has undergone extensive genomic reassortment and mutation, contributing to its evolutionary adaptability. Over the past two decades, highly pathogenic avian influenza (HPAI) viruses, particularly of the A/goose/Guangdong/96-like (GsGd) H5 lineage, have spread globally in multiple epidemic waves [4]. To date, the virus has been detected in over 30 mammalian species, including, for the first time in North America, in ungulates such as dairy cattle and goats [5,6]. The GsGd H5 viruses, which first emerged in China in 1996, have undergone extensive genetic diversification, particularly in the haemagglutinin (HA) gene. The current globally circulating strains fall within clade 2.3.4.4, which is further differentiated by the neuraminidase (NA) gene into subtypes such as H5N1, H5N2, H5N5, H5N6, and H5N8. Since its emergence in 2008, clade 2.3.4.4 has evolved into eight distinct subclades (a–h), with subclade 2.3.4.4b becoming the most prevalent globally [7]. This subclade has been linked to the largest recorded influenza A outbreaks in Europe over the past decade. The 2016–2017 epizootics were predominantly caused by H5N8, which continued to circulate into early 2020. From 2021 onward, H5N1 has become the dominant strain driving infections [8]. In Serbia, H5N1 was first detected in 2006, following the confirmation of HPAI in a mute swan (*Cygnus olor*) carcass [9,10]. This event was part of a wider European epidemic, with cases identified in both wild birds and domestic poultry. In the following decade, H5N1 was not detected in the country, and other HPAI strains, particularly H5N8, became more prevalent. H5N8 caused outbreaks in 2011, re-emerged in 2016, and again in 2021–2022, primarily affecting mute swans. After a 17-year absence, H5N1 re-emerged in Serbia in 2023 and 2024. In 2023, a mass mortality event among common cranes (*Grus grus*) was recorded in the northern part of the country, attributed to HPAI H5N1 of clade 2.3.4.4b [11]. Similar outbreaks were reported in neighbouring countries, suggesting regional spread [10]. In 2024, Serbia confirmed five HPAI outbreaks in wild birds, mainly involving mute swans, and one outbreak in extensively reared domestic poultry [12]. Despite ongoing HPAI surveillance in wild birds, there is a notable lack of genomic data on strains circulating in extensively reared poultry in Serbia, particularly regarding their relationship to wild bird reservoirs. This gap limits our understanding of the viral transmission dynamics at the wildlife–domestic interface and the potential risk of spillover into commercial systems or humans. In Serbia, backyard and extensively reared poultry represent 55% of the poultry sector, yet the surveillance and biosecurity measures in these systems are limited compared to intensive production [13]. In light of these concerns, there is a pressing need to investigate the genetic characteristics of avian influenza viruses in poultry populations that may serve as a bridge between wild reservoirs and commercial flocks.

In this study, we aimed to perform a comprehensive genomic characterisation of influenza A strains identified in backyard chickens and examine their phylogenetic and phylogeographic relationships with both historical and currently circulating strains from wild birds in Serbia and the broader region, seeking to gain deeper insight into their epidemiological significance.

## 2. Materials and Methods

### 2.1. Sample Collection

As part of the national monitoring, seven carcasses of diseased domestic chickens were brought in September 2024 to the Institute of Veterinary Medicine of Serbia after displaying clinical signs indicative of influenza A virus infection. The carcasses originated from a small backyard holding with low biosecurity measures, where the chickens were kept primarily for household use. The date and geographic location were recorded at the time of sampling. A gross pathological examination was conducted, and internal organs were collected for influenza A detection.

### 2.2. Sample Preparation

A 1 cm^3^ sample of pooled tissue was homogenised using a pestle and mortar, then diluted at a 1:10 ratio in Dulbecco’s modified Eagle medium (DMEM; Thermo Fisher Scientific, Waltham, MA, USA) supplemented with antibiotics and antimycotics (MycoZap; Lonza Bioscience, Basel, Switzerland). The homogenate was subsequently centrifuged at 1500× *g* for 10 min, and the resulting supernatant was decanted, passed through a 0.45 μm filter (MF-Millipore; Darmstadt, Germany), and stored at −80 °C until further analysis.

### 2.3. Extraction of Nucleic Acid and qRT-PCR

The total viral nucleic acids were isolated using the IndiSpin Pathogen Kit (Indical Bioscience GmbH, Leipzig, Germany), in accordance with the supplier’s guidelines. To reduce the host DNA content prior to extraction, Turbo DNase (Applied Biosystems, Thermo Fisher Scientific, Waltham, MA, USA) was added to the sample following the manufacturer’s instructions. To monitor the extraction success and serve as an internal quality control, each sample was supplemented with VetMax™ Xeno™ Internal Positive Control RNA (Applied Biosystems, Thermo Fisher Scientific, Waltham, MA, USA). For the detection of influenza A virus RNA, a 20 μL reaction mixture was prepared using the Luna Universal Probe One-Step RT-qPCR Kit (New England BioLabs, Ipswich, MA, USA). The mix included 10 μL of reaction buffer, 0.8 μL of each previously published primer (10 μM), 0.4 μL of probe (10 μM), 1 μL of RT Enzyme Mix, 5 μL of extracted RNA, and 1 μL of Xeno Internal Positive Control RNA primer (Applied Biosystems, Thermo Fisher Scientific, Waltham, MA, USA) [14]. The remaining volume was adjusted with nuclease-free water (RT-PCR Grade Water, Thermo Fisher Scientific, Waltham, MA, USA). An external positive control, consisting of a previously confirmed influenza-A-positive sample, was included in each run to validate both the extraction and amplification steps. This control was diluted to yield a Ct value of approximately 32, which was used as a threshold for interpretation. Nuclease-free water served as the negative control for both extraction and amplification. Thermal cycling was performed according to the kit manufacturer’s recommendations, with an initial reverse transcription at 45 °C for 10 min, followed by enzyme activation at 95 °C for 10 min, and then 40 amplification cycles of 95 °C for 60 s and 60 °C for 45 s. Positive samples were submitted to the National Reference Laboratory at the Veterinary Specialist Institute Kraljevo for influenza virus subtype characterisation.

### 2.4. Isolation on MDCK Cell Line

Virus isolation was performed using the Madin–Darby Canine Kidney (MDCK) cell line. Cells were seeded into 24-well plates (Sarstedt; Nümbrecht, Germany) and incubated at 37 °C with 5% CO_2_ until reaching approximately 80% confluency. Once confluence was achieved, the monolayers were washed twice with DMEM containing 0.8% trypsin (Trypsin–EDTA (0.25%), Thermo Fisher Scientific, Waltham, MA, USA), prepared without foetal bovine serum (FBS). Each well was then inoculated with 0.1 mL of the filtered tissue homogenate, followed by the addition of 1 mL of the 0.8% trypsin–DMEM solution. A mock-inoculated control well was included on each plate to monitor for non-specific cytopathic effects. The cultures were observed daily under an inverted microscope for the appearance of cytopathic effect (CPE) for seven days. At the end of this period, the cells were frozen at −80 °C and subsequently passaged onto fresh MDCK monolayers, with a maximum of three blind passages performed to enhance virus recovery.

### 2.5. Metagenomic Sequencing and Bioinformatic Analysis

Metagenomic libraries were prepared using the SISPA protocol [15]. In Round A, 11 μL of nucleic acid was mixed with 1 μL dNTPs (10 mM) and 1 μL FR26RV-N primer (50 µM), incubated at 65 °C for 5 min, then cooled. A second mix containing 4 μL SSIV buffer, 1 μL SuperScript IV, 1 μL DTT (100 mM), and 1 μL RNaseOUT was added. The combined reaction was incubated at 23 °C for 10 min, then 50 °C for 50 min, and 80 °C for 10 min, followed by 1 μL Klenow polymerase at 37 °C for 1 h, and 75 °C for 10 min. In Round B, the amplification used the FR20RV primer (10 µM) in a 50 μL reaction: 25 μL PfuUltra II mix, 1 μL primer, 3 μL cDNA, and 21 μL RNase-free water. Cycling: 98 °C for 30 s, then 25–35 cycles of 98 °C for 10 s, 65 °C for 30 s, 72 °C for 40 s, and a final step at 72 °C for 2 min. The products were quantified using the Qubit dsDNA HS Assay (Thermo Fisher Scientific, Waltham, MA, USA). Sequencing was performed on the MinION Mk1C (ONT, Oxford, UK) with R10.4.1 flow cells and the Rapid PCR Barcoding Kit (SQK-RPB114.24, ONT, Oxford, UK). The runs lasted 12 h, basecalling, demultiplexing and sequence assembly were performed as previously described [16]. The Nextclade platform was used for clade determination and the sequences were submitted to the NCBI GenBank [17].

### 2.6. Phylogenetic and Phylogeographic Analysis

Sequence alignment was conducted using the MAFFT algorithm in conjunction with 263 hemagglutinin (HA) gene sequences of influenza A H5N1 viruses retrieved from the GISAID EpiFlu™ database (Table S1) [18]. The HA gene sequences originated from various countries across the Northern Hemisphere, including Europe, Asia, and North America. In addition to our isolate, three previously reported H5N1 sequences from Serbia were included in the analysis, one collected in 2023 and two in 2021. The aligned sequences were analysed using Geneious Prime 2025.1.2 (Dotmatics, Boston, MA, USA) and subsequently employed in the phylogenetic and phylogeographic analyses, as previously described [19]. Phylogenetic reconstruction was performed on the IQ-TREE web server using the maximum likelihood (ML) method with ultrafast bootstrap approximation based on 1000 replicates [20]. The resulting ML phylogenetic tree was visualised using FigTree v1.4.4.

Bayesian phylogenetic analysis was conducted using BEAST X v10.5.0 with the BEAGLE v4.0.0 computational library [21]. Multiple Markov chain Monte Carlo (MCMC) runs were carried out under the SRD06 nucleotide substitution model, each with 200 million iterations and a sampling frequency of every 20,000 steps. Marginal likelihood estimation (MLE) was used to assess the model fit across the MCMC runs. The uncorrelated lognormal relaxed molecular clock model and the Bayesian SkyGrid coalescent prior were selected as the best-fitting models based on the Tracer v1.7.2 and MLE values [22]. Maximum clade credibility (MCC) trees were generated using TreeAnnotator v10.5.0, with a 10% burn-in, and visualised in FigTree v1.4.4.

Continuous phylogeographic reconstruction was conducted using a Brownian random walk diffusion model to infer the spatial dynamics over time [23]. Visualisation of the resulting spatiotemporal diffusion patterns was performed using SPREAD3 v.9.6 with the HPD set at 80% [24].

## 3. Results

### 3.1. Virus Isolation and Sequencing

Five out of seven examined carcasses tested positive for influenza A virus by qRT-PCR, with the Ct values ranging from 20 to 31. Subtyping analysis confirmed that all five positive samples were of the H5N1 subtype. These samples were further subjected to virus isolation using the MDCK cell line. Cytopathic effect was observed in one sample, A/chicken/Serbia/01-6035/2024, after the second passage, while the remaining four did not exhibit CPE after three consecutive passages.

The isolate exhibiting CPE was selected for whole genome sequencing. Sequencing yielded 112,520 passed reads, with an average quality score of 10, resulting in one high-quality genome sequence of 13,319 nucleotides in length. The lengths of the eight genomic segments of the isolate were as follows: PB2 2 292, PB1 2 289, PA 2 205, HA 1 704, NP 1 540, NA 1 422, M 998 and NS 869 nucleotides. Complete sequences of all eight genomic segments were submitted to the NCBI GenBank database under accession numbers PV653842 to PV653849. Clade assignment using the Nextclade platform identified the isolate as belonging to clade 2.3.4.4b.

### 3.2. Results of Phylogenetic and Phylogeographic Analysis

Maximum likelihood phylogenetic analysis of the HA gene sequences revealed distinct clustering patterns among the Serbian H5N1 isolates. All the Serbian sequences were grouped exclusively with other European strains, forming a clear separation from those of Asian and North American origin. The newly characterised Serbian isolate clustered with European sequences collected between 2021 and 2025, showing the highest genetic similarity to Hungarian isolates from 2024 and 2025. Notably, A/common_crane/Serbia/12661/2023 clustered within the same group as the strain sequenced in this study, showing close genetic similarity to strains from Spain, Slovenia, and Bulgaria (A/duck/Bulgaria/1220_24VIR2991-2/2023, A/laying-hen/Bulgaria/1619_24VIR11025-15/2024). In contrast, A/swan/Serbia/19524-21/2021 and A/chicken/Serbia/22337-21/2021 formed a separate cluster with earlier European strains, primarily from 2021 and 2022, and shared genetic similarity with sequences from the Czech Republic, Switzerland, Spain, and Croatia (Figure S1).

Bayesian phylogeographic analysis revealed evidence of three independent introductions of the H5N1 influenza virus into Serbia. The earliest two Serbian isolates, A/swan/Serbia/19524-21/2021 and A/chicken/Serbia/22337-21/2021, clustered together with high posterior support. The most closely related sequences were from the Czech Republic, Switzerland and Spain, with their most recent common ancestor (MRCA) dated to January 2021. This suggests a single introduction followed by local transmission during early 2021. The MRCA of the 2021 isolates and the more recent 2024 isolate were dated to November 2020, implying a common ancestral lineage. In contrast, the MRCA of the 2024 isolate A/chicken/Serbia/01-6035/2024 and A/common_crane/Serbia/12661/2023 was estimated to have emerged around December 2022, indicating divergence from the 2021 cluster and supporting the occurrence of a separate introduction event (Figure S2).

**Figure 1:**
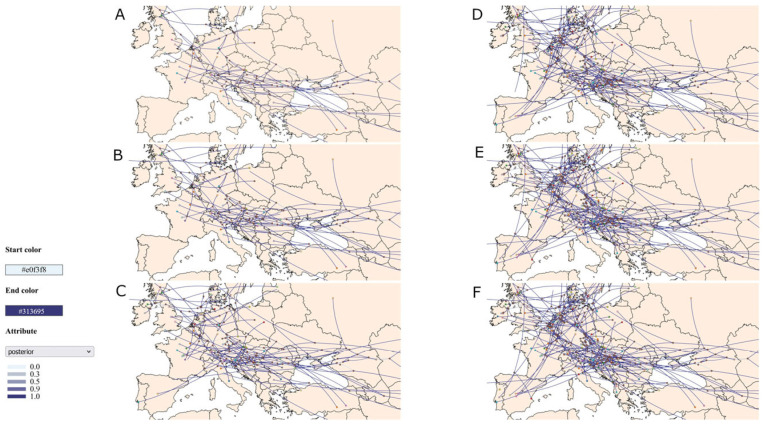
Spatiotemporal spread of H5N1 influenza virus in Europe, 2020–2025. Maps (**A**–**F**) display the inferred diffusion pathways of the H5N1 influenza virus in Europe for each year from 2020 (**A**) to 2025 (**F**). The lines represent viral dispersal pathways, with the colour intensity indicating the posterior probability. The line colour ranges from lighter (#e0f3f8) to darker (#313695), reflecting the level of statistical support: light lines indicate lower support, and darker lines indicate higher support. The brown dots mark internal nodes, representing the inferred ancestral locations.

Spatiotemporal analysis using SPREAD3 confirmed three independent introductions of H5N1 influenza into Serbia between 2020 and 2024. The majority of the key migration nodes showed posterior probabilities above 0.9, indicating strong support for the inferred transmission pathways based on the MCC tree (Figure S2). In 2021, two sequences were reported in Serbia, one in February and the other in April, both tracing back to a common ancestor estimated to have emerged in January 2021 near the Serbia–Croatia border. The analysis suggested that the strain A/chicken/Serbia/22337-21/2021 may have diverged in Romania later in 2021, giving rise to the strain A/swan/Romania/16905_22VIR2749-1/2021. In November 2023, a new H5N1 virus was detected in Serbia, showing the closest genetic relationship to an isolate from Spain, with a common ancestor located in Italy in September 2023. The H5N1 virus characterised in this study was detected in Serbia in September 2024. Spatiotemporal analysis indicated that the virus likely originated in Hungary, sharing a common ancestor with Hungarian strains from 2024 and 2025, with the most recent common ancestor dated to December 2023 (Figure 1).

In addition to the viruses detected in Serbia, spatiotemporal analysis using SPREAD3 revealed several divergent events within Serbian territory with strong statistical support (posterior > 0.9), not directly related to the viruses detected in the country. In late August and September 2021, two internal nodes were inferred to have emerged from Central Europe into northern Serbia. One of these nodes was identified as the potential ancestral node of the strain detected at the end of 2021 in Bulgaria (A/duck/Bulgaria/756-4_22VIR778-6/2021), while the other represented the ancestral node of the strain identified in Romania (A/swan/Romania/16905_22VIR2749-1/2021). In August 2023, an internal node appeared in western Serbia, descending from A/mute_swan/Bosnia_and_Herzegovina/213/2021. This node is the likely ancestor of three sequences: two from Bulgaria (A/duck/Bulgaria/1220_24VIR2991-2/2023, A/laying-hen/Bulgaria/1619_24VIR11025-15/2024) and one from North Macedonia (A/captive-goose/Macedonia/MKD_G_ZOO_24VIR10980-1/2024). A third internal node, although supported by a lower posterior probability, suggested a divergent event from Central Europe in eastern Serbia in June 2024. This node is a possible ancestral origin (posterior > 0.9) of the virus detected in Romania in January 2025 (A/whooper-swan/Romania/10165_25VIR1011-7/2025) (Figure 1).

## 4. Discussion

This study investigated the phylogenetic and phylogeographic patterns of H5N1 influenza viruses circulating in Serbia, including newly isolated and previously reported strains. The recent emergence of the H5N1 subtype belonging to clade 2.3.4.4b led to a significant outbreak in Serbia in 2021, and the virus has been detected annually in the country since. Our phylogeographic analysis revealed three distinct introductions of clade 2.3.4.4b H5N1 into Serbia since 2021. In all three cases, the virus was likely introduced from Central or Western Europe.

Our study showed that the Serbian strains from early 2021 were genetically similar to Central European strains, and spatiotemporal analysis suggested that their ancestral node was likely located in the Western Balkans, indicating a Central European origin followed by local divergence and subsequent eastward spread into Romania. In 2021, two ancestral nodes of the H5N1 strain from Bulgaria and Romania were identified in northern Serbia. During the 2020–2021 and 2021–2022 epidemic waves, North–Central Europe was identified as the primary source of the H5N1 virus introduction to the rest of the continent. These incursions acted as key entry points for the subsequent spread of the virus across Europe, including into the Western Balkans and, to a lesser extent, the Eastern Balkan countries. Local west-to-east transmission within the Balkan Peninsula was also observed during these epidemic periods [8].

A similar spatial pattern of viral spread was observed in our study during the period 2022–2025. Spatiotemporal analysis revealed that Serbia represents an important site of viral divergence between Western and Eastern Europe. We identified a potential ancestral node between the 2023 Serbian strain and a genetically similar strain from Spain, with the most recent common ancestor located in Italy in September 2023. A previous study from Serbia describing the isolate A/common_crane/Serbia/12661/2023 concluded that it was closely related to Croatian and Italian strains, suggesting migratory common cranes as a likely source of introduction [11]. Furthermore, our spatiotemporal analysis identified a key internal node in western Serbia in 2023, from which multiple divergent events emerged, indicating viral spread from Bosnia and Herzegovina into Serbia and further eastward into Bulgaria and North Macedonia. Interestingly, maximum likelihood phylogenetic analysis showed that the Bulgarian strains diverging from this node were genetically similar to the Serbian virus detected in 2023. Additionally, ML analysis indicated that the strain from Bosnia and Herzegovina grouped with the Serbian strains from 2021. Our spatiotemporal analysis suggested that the ancestor of the H5N1 virus detected in Bosnia and Herzegovina likely originated in Croatia, with divergence estimated to have occurred in September 2021. This strain from Bosnia and Herzegovina sampled in November 2021 from a mute swan near the Croatian border along the Sava River, carried the D473N substitution, which was also identified in a virus from Croatia (A/mute swan/Croatia/100/2021) and one from the Netherlands (A/goose/Netherlands/21039029-001/2021) [10]. The A/mute_swan/Croatia/100/2021 strain from Croatia was sampled during the same period, also from a mute swan, and clustered closely with the Bosnian strain. However, the authors did not include the H5N1 Serbian strains from 2021 in the research. These findings confirm the complex distribution and transmission dynamics of the H5N1 influenza virus in the Balkan Peninsula, characterised primarily by west-to-east spread across the region.

Our results suggest that the new H5N1 isolate, A/chicken/Serbia/01-6035/2024, is closely related to strains circulating in Hungary and was most likely introduced from that region. Between September and December 2023, Hungary reported 43 H5N1 outbreaks in poultry, whereas no outbreaks were reported in Serbia during this period [25]. Concurrently, 35 detections of the virus were reported in wild birds in Hungary and 10 in the northern region of Serbia. In Hungary, the virus was predominantly detected in common cranes, whereas in Serbia, the virus was detected in both common cranes and waterfowl. During the same period, Croatia reported a single outbreak in domestic poultry and one detection in wild birds, while no cases were reported in Bosnia and Herzegovina. Between March and June 2024, poultry outbreaks in Europe were reported exclusively in Hungary and Bulgaria [26]. Notably, only a single H5N1 detection was recorded in Hungary, again in a common crane. These epidemiological data support our phylogeographic findings, which indicate that the 2024 Serbian strain is closely related to Hungarian H5N1 viruses and was most likely introduced from Hungary in late 2023.

These findings highlight the recurring role of Serbia as both a recipient and a transit point in the regional spread of H5N1 across the Balkans. The migration of wild birds is likely one of the most influential factors in virus transmission, particularly in the Balkan Peninsula. Water bodies, like the Sava and Danube rivers, serve as key stopover and overwintering habitats for many bird species. Among wild birds, common cranes and various species of wild waterfowl play a central role in regional spread and act as both reservoirs and environmental vectors of the virus. Domestic fowl and waterfowl, particularly those belonging to the family Anseriformes, also exhibit high genetic diversity and contribute to viral maintenance and reassortment [8]. Following the introduction of H5N1 into Europe, nearly every subsequent transmission event across the continent has been correlated with wild bird migration. Seasonal migration and periodic contact between wild birds and domestic poultry are considered key mechanisms in the emergence and dissemination of novel strains [27]. There are four main migratory routes of the common crane in Europe: the Western European, Eastern European, Baltic–Hungarian, and Volga–Caucasian flyways. Birds following the Baltic–Hungarian flyway often stop in eastern Hungary and northern Serbia, with some overwintering in these regions, while others continue to Africa or Italy. These birds typically remain in the northern regions from early spring through autumn for the breeding season, before migrating southward in autumn. During migration, they stop in ecologically favourable areas to rest and refuel [28]. Similar to common cranes, wild waterfowl also migrate from north to south in autumn along well-defined flyways. One of the major migratory routes, the Black Sea/Mediterranean flyway, connects Northern Europe and Asia with Africa and intersects the Balkan Peninsula. Notably, although the spring and autumn migrations follow similar routes, it seems that the autumn southward migration plays a more significant role in the transmission of H5N1 [29].

While our findings contribute to a better understanding of the molecular epidemiology and transmission dynamics of H5N1 viruses in Serbia and the broader Balkan region, these conclusions should be interpreted with caution due to certain limitations of this study. The relatively small number of available samples and full genome sequences may restrict the resolution of the phylogenetic relationships. The reliance on passive surveillance, particularly in backyard flocks, also introduces potential sampling bias that could underestimate the true diversity and circulation patterns of H5N1 viruses in the region.

## 5. Conclusions

Spatiotemporal analysis confirmed both the north–south and west–east dissemination patterns of H5N1 influenza virus from Northern and Central Europe into the Balkan Peninsula. The analysis further revealed a west–east transmission route within the Balkans, with Serbia serving as a key transit point. Geographically, Serbia is centrally located in the Balkan Peninsula and intersected by two major rivers, the Sava and the Danube, which are important ecological corridors for migratory birds. Given that several major avian migratory routes cross through Serbia, the country experiences significant seasonal bird movement involving various species. This creates opportunities for viral exchange between migratory birds, local wild bird populations, and domestic poultry, particularly backyard and extensively reared flocks kept under low biosecurity conditions, which may come into direct or indirect contact with wild birds. However, the limited availability of genomic data and surveillance reports from Serbia and neighbouring countries such as Bosnia and Herzegovina, Montenegro, and North Macedonia represents a substantial knowledge gap. Future research should prioritise investigations into the local transmission dynamics and host-specific patterns of H5N1 influenza virus spread within the Balkan region to better understand its epidemiology and inform targeted surveillance and control strategies.

## Data Availability

The data presented in this study are available on request from the corresponding author.

## Supplementary Materials

The following supporting information can be downloaded at https://www.mdpi.com/article/10.3390/pathogens14070636/s1, Table S1: Sequence metadata, Figure S1: ML tree, Figure S2: MCC tree.
